# Pioneers in neurology: Charles Miller Fisher (1913–2012)

**DOI:** 10.1007/s00415-021-10760-x

**Published:** 2021-09-22

**Authors:** Konrad Kubicki, Andrzej Grzybowski

**Affiliations:** 1 Department of Neurological Sciences, Rush University Medical Center, Professional Building, 1725 W Harrison St, Suite 1106, Chicago, IL 60612 USA; 2 Department of Ophthalmology, University of Warmia and Mazury, Olsztyn, Poland; 3 Institute for Research in Ophthalmology, Gorczyczewskiego 2/3, 61-553 Poznan, Poland

Charles Miller Fisher was born on December 5, 1913, in Waterloo, Ontario, Canada. He completed his medical degree at the University of Toronto in 1938. He was awarded a competitive internship at Henry Ford Hospital in Detroit, Michigan, subsequently starting residency at the Royal Victoria Hospital in Montreal [1]. In the backdrop of WWII, C M Fisher volunteered for the Canadian Navy in 1940; however, after the fall of France, he was transferred to the British Royal Navy on loan due to the urgent need for more naval medical officers. He served as the physician of an armed cruiser named the *Voltaire* that was attacked by a German raider in 1941. Rescued from the ocean after six hours by the enemy, he became a prisoner-of-war at German camps for over 3 years [1]. His meticulous attention for detail is evident from his first publication in 1945—an account of medical observations and treatments provided in these camps [2].

After repatriation in September 1944, C M Fisher returned to Canada for a refresher course in medicine which included a 2-month rotation at the Montreal Neurological Institute (MNI). It was here, under neurosurgeon and MNI director Dr. Wilder Penfield that his career shifted toward neurology. Having impressed the institute director with accurately diagnosing the cause and location of a patient’s focal seizures, an acting-registrar position at MNI was arranged. Encouraged to continue with postgraduate training abroad, a yearlong neuropathology fellowship at Boston City Hospital under Dr. Raymond Adams became the next destination in 1949 [1]. Almost immediately, he made pivotal observations from post-mortem examinations. Specifically, atrial fibrillation had not been previously associated with stroke. C M Fisher demonstrated the concept that atrial emboli migrate, cause infarction, undergo lysis with subsequent reperfusion, and result in secondary petechial hemorrhages [3]. This novel hypothesis was not accepted by pathology journals when it was first proposed, making him realize that little was known about vascular pathology in cerebrovascular diseases [1].

C M Fisher returned to Montreal in 1950 to become the neuropathologist at Montreal General Hospital and continued making instrumental observations. This was followed by an invitation in 1954 from Dr. Adams, then newly appointed Chief of Neurology at Massachusetts General Hospital (MGH), to return to Boston with the aim of developing the first ever stroke service [1]. This set forth a collaboration which helped change the face of neurology, with interest in the research and care of stroke patients lifted out of obscurity. Under C M Fisher, the service produced trainees that became worldwide leaders in stroke care; moreover, stroke became recognized as, primarily, a neurological discipline. Receiving numerous accolades for having revolutionized the clinical and neuropathological understanding—and management—of cerebrovascular diseases, he especially treasured induction into the Canadian Medical Hall of Fame in 1998 [4]. He credited his selfless wife, Doris, for managing all nonmedical aspects of his life—often driving him to work and back, after the late nights spent at MGH toward the later years of his career. Although officially retired from Professorship at MGH in 1980, he nonetheless continued to be remarkably active over the next 25 years as Emeritus Professor at Harvard Medical School and Senior Consultant. Doris and C M Fisher—married for 68 years—were survived by two sons and a daughter. He passed on April 14, 2012, in Albany, New York at the age of 98 due to an accumulation of ailments [4].

One foundational contribution from early on in his career, in 1951, includes the reported association between stroke and carotid artery disease [6]. Removal of 1100 pairs of carotid arteries supported his hypothesis [5], and this opened the door therapeutically to carotid endarterectomy for surgeons who were starting to operate on the peripheral vascular system. C M Fisher reported the prodromal symptoms of these patients with extracranial carotid disease and suggested the concept that these spells were ischemic in nature—as opposed to vasospastic, as previously argued—coining the term transient ischemic attack (TIA) [5]. His lasting impact on the management of these patients is evident from the idea of anticoagulating patients presenting with TIA or ischemic stroke to prevent a subsequent stroke [7]. In addition to atrial fibrillation and carotid stenosis, he uncovered carotid artery dissection as another cause of stroke [8].

C M Fisher’s methodical approach and exceptional observational skills are reflected in his extensive descriptions of 20 different lacunar stroke syndromes, published in 1982 [9]. These include clinical features, etiologies, radiological findings, and clinicopathological correlations in pure sensory stroke, pure motor hemiparesis, ataxic hemiparesis, dysarthria-clumsy hand syndrome, etc. Meticulously referring to case reports, he argued against the accepted notion of a “lacunar state” with haphazardly located lacunes (instead, more accurately attributing normal-pressure hydrocephalus to cited cases) [9]—demonstrating his propensity for questioning dogma after carefully analyzing the available data. Moreover, despite sharing caution over the growing reliance on neuroimaging, he continuously strived toward innovation and proposed the Fisher score as a metric of aneurysmal subarachnoid hemorrhage severity with subsequent risk of vasospasm. Based on computed tomographic evidence of blood volume distributed in the subarachnoid space, this contribution augmented the recognition of vasospasm as a sequela of subarachnoid hemorrhage [10].

Other notable cerebrovascular disease contributions include describing clinicopathological features of thalamic and cerebellar hemorrhage, lateral medullary infarction, basal rupture of intracranial aneurysm, inflammatory vascular disease, and reversible cerebral vasospasm [1, 5]. With a bibliography of over 200 publications, his contributions to the field of general neurology are likewise impressively significant. This includes describing clinical presentations of the following: normal-pressure hydrocephalus, transient global amnesia, the variant of Guillain–Barre syndrome known as Miller Fisher syndrome, ocular bobbing, one-and-a-half syndrome, and the rostral-caudal deterioration of comatose patients [1, 4]. As such, C M Fisher is to be credited with playing a major role in advancing the field of neurology. An astute observer, resolute investigator, phenomenal clinician, and cherished mentor, his everlasting passion for neurology was evident by the writing of articles well into his nineties. This fantastic exemplar of steadfast dedication to improving the lives of patients will continue to serve as a beacon, inspiring current and future generations of clinician-investigators (Fig. 1).

**Fig. 1:**
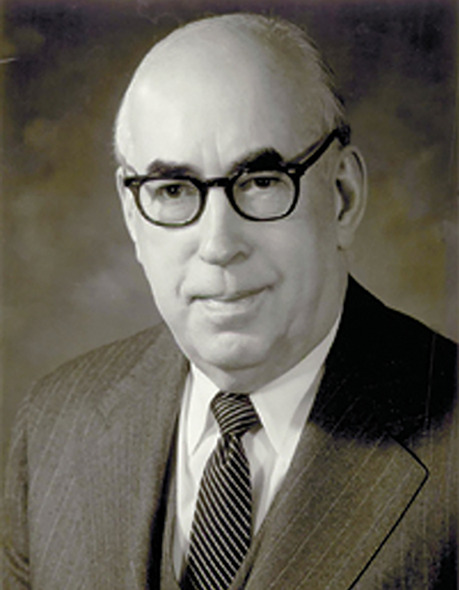
Charles Miller Fisher (1913–2012). Public domain. Source: https://alchetron.com/C.-Miller-Fisher

## Data Availability

Not applicable.
